# First-In-Human Study in Cancer Patients Establishing the Feasibility of Oxygen Measurements in Tumors Using Electron Paramagnetic Resonance With the OxyChip

**DOI:** 10.3389/fonc.2021.743256

**Published:** 2021-10-01

**Authors:** Philip E. Schaner, Benjamin B. Williams, Eunice Y. Chen, Jason R. Pettus, Wilson A. Schreiber, Maciej M. Kmiec, Lesley A. Jarvis, David A. Pastel, Rebecca A. Zuurbier, Roberta M. DiFlorio-Alexander, Joseph A. Paydarfar, Benoit J. Gosselin, Richard J. Barth, Kari M. Rosenkranz, Sergey V. Petryakov, Huagang Hou, Dan Tse, Alexandre Pletnev, Ann Barry Flood, Victoria A. Wood, Kendra A. Hebert, Robyn E. Mosher, Eugene Demidenko, Harold M. Swartz, Periannan Kuppusamy

**Affiliations:** ^1^ Department of Medicine, Norris Cotton Cancer Center, Geisel School of Medicine at Dartmouth College, and Dartmouth-Hitchcock Medical Center, Lebanon, NH, United States; ^2^ Department of Radiology, Norris Cotton Cancer Center, Geisel School of Medicine at Dartmouth College, and Dartmouth-Hitchcock Medical Center, Lebanon, NH, United States; ^3^ Department of Surgery, Norris Cotton Cancer Center, Geisel School of Medicine at Dartmouth College, and Dartmouth-Hitchcock Medical Center, Lebanon, NH, United States; ^4^ Department of Pathology, Norris Cotton Cancer Center, Geisel School of Medicine at Dartmouth College, and Dartmouth-Hitchcock Medical Center, Lebanon, NH, United States; ^5^ Department of Chemistry, Norris Cotton Cancer Center, Geisel School of Medicine at Dartmouth College, and Dartmouth-Hitchcock Medical Center, Lebanon, NH, United States; ^6^ Department of Biomedical Data Science, Norris Cotton Cancer Center, Geisel School of Medicine at Dartmouth College, and Dartmouth-Hitchcock Medical Center, Lebanon, NH, United States

**Keywords:** OxyChip, oximetry, EPR, tumor, radiation, chemotherapy, hyperoxygenation

## Abstract

**Objective:**

The overall objective of this clinical study was to validate an implantable oxygen sensor, called the ‘OxyChip’, as a clinically feasible technology that would allow individualized tumor-oxygen assessments in cancer patients prior to and during hypoxia-modification interventions such as hyperoxygen breathing.

**Methods:**

Patients with any solid tumor at ≤3-cm depth from the skin-surface scheduled to undergo surgical resection (with or without neoadjuvant therapy) were considered eligible for the study. The OxyChip was implanted in the tumor and subsequently removed during standard-of-care surgery. Partial pressure of oxygen (pO_2_) at the implant location was assessed using electron paramagnetic resonance (EPR) oximetry.

**Results:**

Twenty-three cancer patients underwent OxyChip implantation in their tumors. Six patients received neoadjuvant therapy while the OxyChip was implanted. Median implant duration was 30 days (range 4–128 days). Forty-five successful oxygen measurements were made in 15 patients. Baseline pO_2_ values were variable with overall median 15.7 mmHg (range 0.6–73.1 mmHg); 33% of the values were below 10 mmHg. After hyperoxygenation, the overall median pO_2_ was 31.8 mmHg (range 1.5–144.6 mmHg). In 83% of the measurements, there was a statistically significant (p ≤ 0.05) response to hyperoxygenation.

**Conclusions:**

Measurement of baseline pO_2_ and response to hyperoxygenation using EPR oximetry with the OxyChip is clinically feasible in a variety of tumor types. Tumor oxygen at baseline differed significantly among patients. Although most tumors responded to a hyperoxygenation intervention, some were non-responders. These data demonstrated the need for individualized assessment of tumor oxygenation in the context of planned hyperoxygenation interventions to optimize clinical outcomes.

## Introduction

Most solid tumors contain regions of acute and chronic hypoxia that can negatively impact treatment outcomes in cancer patients (1–7). The use of the Eppendorf technique and other modalities has demonstrated that the pre-treatment oxygen levels in solid tumors are a critical parameter affecting clinical outcomes, particularly using radiation therapy, as hypoxia causes resistance to treatment (1–6). Evidence of poor outcomes for hypoxic tumors is particularly strong for squamous cell carcinomas of both the head and neck (8–14) and cervix (15, 16). The results from the ARCON (accelerated radiotherapy combined with carbogen and nicotinamide) trial in head-and-neck cancer (17) emphasize in particular the need for a stratification of this patient population with respect to tumor hypoxia in order to optimize treatment outcomes. In a subset of patients who participated in a translational side study, a histologic marker of hypoxia (pimonidazole) was used to analyze biopsy specimens. This subset analysis revealed that ARCON improved both regional control and disease-free survival in the group of patients with hypoxic tumors, while the group of patients whose tumors did not have hypoxia (as defined by localization of pimonidazole) did not benefit from the ARCON protocol. These data emphasize the need for individualized oxygen-based stratification of patients to evaluate the efficacy of hyperoxygenation interventions to enhance therapeutic outcomes (17). Therefore, it is highly desirable to monitor oxygen levels in tumors before, during, and after therapeutic interventions. This would require the availability of a means to make reliable, repeated, and direct measurements of oxygen levels in tissue at specific anatomical locations—a capability that presently is not available in the clinic. Consequently, interventions to address tumor hypoxia have not been widely integrated into the standard of care (SOC) practice of clinical radiotherapy, nor into any other cancer treatments that might benefit from such interventions.

While there are several clinically viable techniques that can directly assess tumor hypoxia including polarographic electrodes, fluorescence-quenching, and direct injection of oxygen-sensitive NMR probes based on fluorine (18–22), these techniques have the disadvantage of not being able to be used repeatedly or routinely (23). BOLD MRI, proton NMR spectroscopy, DWI MRI, duplex Doppler ultrasound, PET based on metabolism of hypoxia-localizing drugs (24–26), and near-infrared (NIR) measurements of hemoglobin are widely available non-invasive techniques that assess tissue oxygenation. However, these methods provide data on parameters that, while related to tissue oxygen, do not directly measure oxygen in the tumors, and their relationship to tumor oxygen has yet to be established. In contrast, *in vivo* EPR oximetry can make clinically relevant dynamic measurements of tissue oxygen levels with the unique capability to perform repeated measurements over time (27–35). EPR oximetry relies on an oxygen-sensing paramagnetic probe implanted within a tissue of interest to measure partial pressure of oxygen (pO_2_) surrounding the probe. Following implantation of the probe oxygen measurements are obtained non-invasively by placing a surface-coil detector over the probe, and pO_2_ measurements are then made in real time as often as desired (36).

While initial *in vivo* EPR oximetry measurements were made using soluble probes and carbon particulates (37), crystalline materials such as lithium phthalocyanine (38) or naphthalocyanine (39) and derivatives (40, 41) are ideally suited for EPR oximetry. While all of these materials are highly EPR sensitive and measure tissue oxygen (pO_2_) directly, the crystalline material has favorable spectroscopic properties including narrow line shape and high spin density. The crystals can be easily embedded in biocompatible polymers to prevent interactions with tissues and ensure their structural integrity. There is a linear relationship between the EPR spectral width and the pO_2_ surrounding the crystalline material; moreover, measurements of pO_2_ are particularly sensitive at levels of hypoxia, which are of greatest clinical significance.

One such oxygen-sensing paramagnetic material is lithium octa-butoxynaphthalocyanine (LiNc-BuO) crystals (40, 42). An implantable oxygen probe, called the OxyChip, has been developed by embedding LiNc-BuO crystals in the oxygen-permeable polymer polydimethylsiloxane (PDMS) for pO_2_ measurements (43–47). Embedding the LiNc-BuO in PDMS shields the crystals from interaction with the biological micro-environment, thereby preventing biochemical degradation and breakdown, as well as local and/or systemic interactions. Embedding can also preserve the localization and quantity of the crystals once implanted. The probe can be removed, for example during standard-of-care (SOC) en bloc surgical resection of the tumor. Here, we report oxygen data from a first-in-human clinical study on the feasibility of the OxyChip for individualized tissue-oxygen assessment prior to and during hypoxia-modification interventions.

## Patients and Methods

### Patient Eligibility

Patients with solid tumors were recruited and enrolled in the clinical trial “Oxygen Measurements in Subcutaneous Tumors by EPR Oximetry Using OxyChip” (NCT02706197). The study was carried out in accordance with USA and international standards of Good Clinical Practice - FDA Title 21 part 312 and International Conference on Harmonization guidelines. The study protocol (IRB Study 00028499) was approved by the Institutional Review Board (IRB) at Dartmouth College and Dartmouth-Hitchcock Medical Center (DHMC). The Food and Drug Administration (FDA) approved an IDE (G130260) for use of the OxyChip (see below). All subjects gave written informed consent in accordance with the Declaration of Helsinki. Eligibility criteria for patients were: age ≥18-years old, having the capacity to give informed consent in English, having a tumor (benign or malignant) >2.5 cm in diameter (added during a protocol revision), having a tumor at a depth of ≤3 cm from the skin surface, and having a planned surgical resection of the tumor at least three days after implantation as part of SOC therapy. Patients were excluded from this study if they were pregnant, had contraindications for exposure to a magnetic field, had prior radiotherapy to the site of implantation, were to receive angiogenesis inhibitors during the study, or had a platelet blood count of <50,000/µl of blood and an absolute neutrophil count of <1,000/µl of blood. An initial cohort of six patients who received surgery alone after OxyChip implantation was evaluated for safety and toxicity endpoints. After this evaluation demonstrated no significant safety or toxicity findings, a second cohort was opened, in which patients could have either chemotherapy or radiotherapy prior to surgical resection but not both concurrently or consecutively.

### *In Vivo* EPR Oximetry With the OxyChip

**Figure 1:**
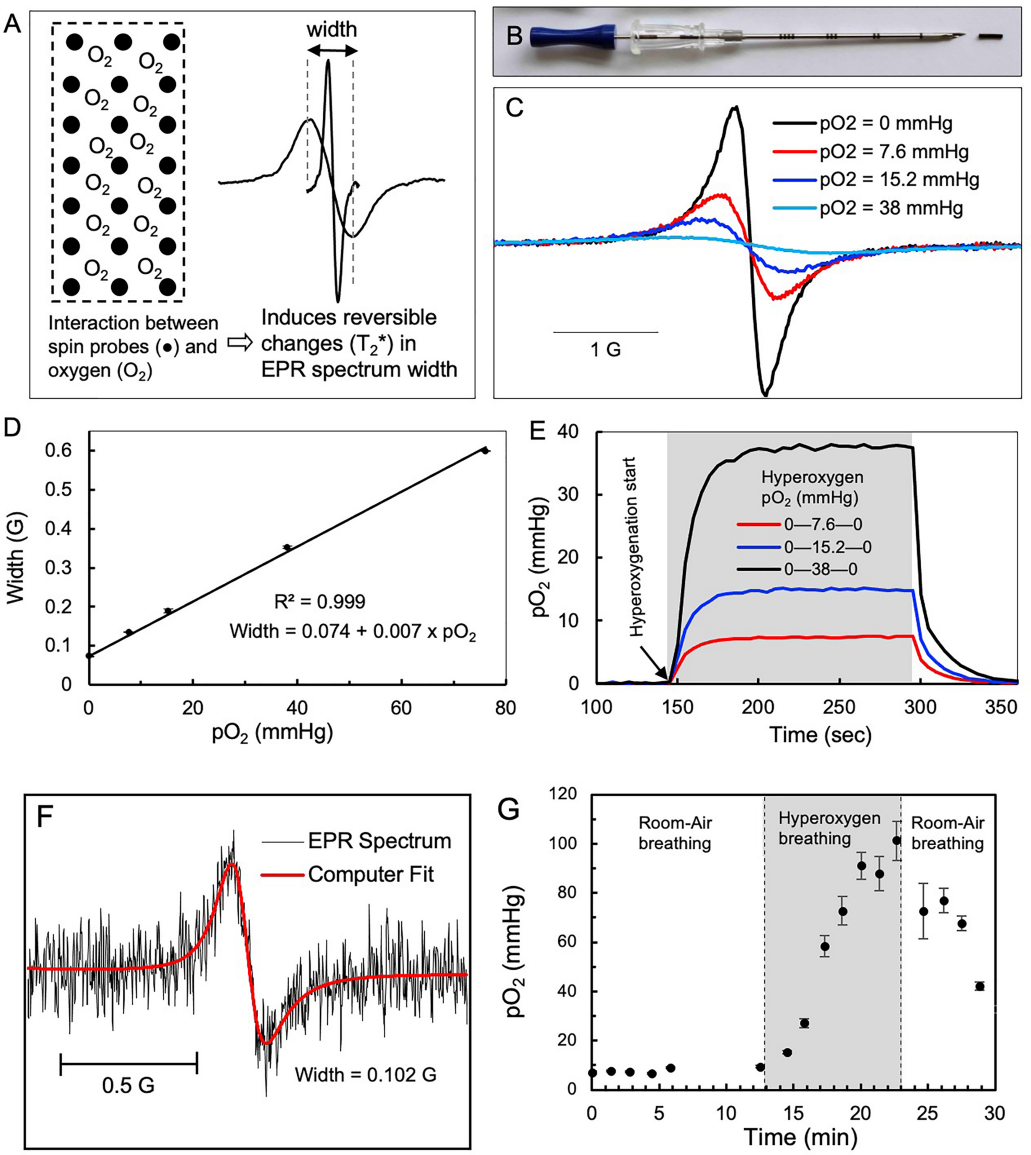
EPR oximetry using OxyChip. Oxygen (pO_2_) measurements in tumors were made by EPR oximetry using the OxyChip. **(A)** Illustration of the principle of EPR oximetry using the OxyChip. **(B)** Photograph showing an OxyChip along with a brachytherapy needle used for implantation in tumors. **(C)** EPR spectra of an OxyChip obtained *in vitro* in the presence of different oxygen levels (pO_2_ in mmHg): 0, 7.6, 15.2, and 38 at room temperature. The spectra exhibit oxygen-dependent broadening. **(D)** EPR width of the OxyChip measured in the pO_2_ range 0–76 mmHg at room temperature. The data (mean ± SD; n=5) exhibit a linear response of EPR width to pO_2_. **(E)** Time-response of the OxyChip to changes in pO_2_ levels obtained *in vitro*. For all pO_2_ levels (in mmHg) baseline measurements were obtained at 0, hyperoxygenation was initiated (gray block: 7.6; 15.2; and 38 mmHg), and then oxygen was discontinued with a return to baseline (0)mmHg. The data indicated a time-response of about 30 sec to reach equilibrium in each case. **(F)** Representative EPR spectrum, computer fitting, and estimated EPR spectral width obtained from the tumor of a patient (20 in **Table 1**). Superimposed in red is a computer fit used to obtain spectral width, which was converted to pO_2_ using a calibration curve **(D)**. **(G)** Representative pO_2_ data obtained from a patient (9 in **Table 1**) during a session of room-air breathing, hyperoxygen breathing (gray block) and return to room-air breathing.

In vivo EPR oximetry refers to measuring oxygen in living tissue by EPR spectroscopy (37). The principle of EPR oximetry is based on the paramagnetic property of molecular oxygen (O_2_), which in its ground state has two unpaired electrons that can undergo spin-exchange interaction with a paramagnetic EPR probe (**Figure 1A**). This process is sensitive to the partial pressure of oxygen (pO_2_) at the probe location, with the relaxation rate of the probe increasing as a function of pO_2_ in the tissue adjacent to the probe (37, 48). The OxyChip is an oxygen-sensing probe containing paramagnetic LiNc-BuO crystals embedded in PDMS for clinical applications (43–47). The OxyChips used in this study were of a cylindrical shape, 5 mm in length and 0.6 mm in diameter (**Figure 1B**). The EPR spectrum of the OxyChip responds to pO_2_ in a predictable manner (**Figures 1C, D**) and responds quickly (~30 sec) to changes in pO_2_ levels (**Figure 1E**). Each OxyChip used in this study was verified for its oxygen response (calibration) before implantation and after removal from patients.

### OxyChip Implantation

The OxyChip is classified as a Class III medical device by the FDA’s Center for Device and Radiological Health. Based on biological evaluation (ISO 10993-12:2012 guidelines) and preclinical testing data (36), the FDA granted investigational device exemption (IDE) status to the OxyChip for a clinical study to collect safety data associated with the OxyChip and to assess the feasibility of making pO_2_ measurements in tumors, especially in cancer patients (49–51). The OxyChips were steam-sterilized in a clinical autoclave at gravity cycle (set at 121°C/15 PSIG for 30 minutes) with appropriate biological and chemical indicators and stored in a sterile environment prior to implantation. The OxyChip was implanted under sterile conditions by placing it inside an 18-G brachytherapy needle (**Figure 1B** **)** and deploying it into the tumor under local anesthesia (1% lidocaine) unless the patient declined the anesthetic (**Supp. Figure 1**). Ultrasound image guidance was used to direct needle placement where it was deemed necessary for definitive placement of the OxyChip in the tumor. After implantation, patients were evaluated for associated toxicity by a study physician immediately after OxyChip placement, during all EPR oximetry measurements, and, if the patient received chemotherapy or radiotherapy, at all chemotherapy administration appointments and at least weekly for those undergoing radiotherapy. Evaluations also occurred within two weeks of surgical resection of the tumor and patient records were monitored for potential adverse events until a year after OxyChip removal. Adverse events were scored using the Common Terminology Criteria for Adverse Events (CTCAE) v4.0.

### EPR Measurements

EPR oximetry was carried out using a custom-built EPR scanner (**Supp. Figure 2A**) operating at 1.15 GHz (52). Patients were positioned on a gurney within the magnetic field, and the EPR surface-coil detector was placed over the site of the implanted OxyChip (**Supp. Figure 2B**) (53). In some patients, ultrasound imaging was used to locate the OxyChip prior to placement of the detector. After detector (resonator) tuning, and optimization of data-acquisition settings, EPR scans were made. For each scan, the magnetic field was swept over the range of the EPR signal and 1024 data points were collected. The scan period was 5 seconds, and scans were repeated and typically accumulated for 1 minute (i.e., twelve 5-second scans) under non-saturating RF power (**Figure 1F**). Overmodulation was used as needed to improve the signal-to-noise ratio (SNR). This acquisition process was repeated throughout the baseline, hyperoxygenation, and recovery periods. During each measurement session, the patient would breathe room air, followed by a period of oxygen inhalation using a non-rebreather mask with 100% oxygen delivered at a flow rate of 15 liters/minute, and then breathe room air again; all three measurement periods were carried out for up to ten minutes (approximately), for an anticipated total of up to 30 minutes (**Figure 1G**). Not all patients completed all parts of the measurement protocol due primarily to technical or logistical considerations (e.g., the OxyChip signal was not detected or patient time constraints) as opposed to any difficulty tolerating EPR measurements. Measurement sessions were repeated as often as the patient was willing and available at the clinic prior to surgical resection.

### Estimation of pO_2_ Data From EPR Spectra

Median spectra were calculated for each set of consecutive 5-second EPR scans based on the point-by-point median across scans (for 1024 points) and computer-fitted to obtain the width (half-width at half-maximum), which was then converted to pO_2_ using a width-vs-pO_2_ calibration curve established *in vitro* (i.e., **Figure 1D**). Typically, 12 scans per set were acquired and each pO_2_ value corresponds to one minute of EPR data acquisition. The measurement was considered successful if the median spectrum showed the characteristic single-component signal centered at the expected magnetic-field-sweep position corresponding to the OxyChip signal. Although the EPR measurements are known to be stable, occasional involuntary or accidental movement by the patient or resonator would distort the EPR spectral shape making the fitting results unusable, in which case it would be excluded from use. The fitting software models the effects of overmodulation as reported by Robinson et al. (54, 55) to extract the intrinsic linewidth through curve fitting of the over-modulated spectra. The baseline pO_2_ values reported herein are mean ± SEM (standard error of the mean) of the pO_2_ values obtained during the time the patient breathing room air, before switching to hyperoxygen breathing. The hyperoxygenation pO_2_ values reported herein represent the pO_2_ values (±SEM) estimated at the end of 10 minutes after switching to hyperoxygen breathing, regardless of actual duration of hyperoxygenation. These values were calculated from a linear fitting of the pO_2_ values obtained during hyperoxygen-breathing (**Supp. Figure 3**). In this way, we could express the hyperoxygen pO_2_ data on a uniform timescale. The details of the pO_2_ values or dynamics during the recovery period (back to room-air breathing) have not been analyzed and are not discussed in this report.

### Characterization of Explanted OxyChips

The OxyChips were removed as part of the en bloc tumor resection during SOC surgery. This information is reported in detail in a separate report (51). Both gross and microscopic evaluations of the tissue surrounding the OxyChip were performed. The integrity of the OxyChip (shape, length), its placement relative to tumor location, and the distance from the skin surface were grossly assessed in the pathology laboratory. The location of the OxyChip relative to the tumor was described and was identified as outside the tumor if non-tumor cells surrounded it.

### Statistical Analyses

Significant differences between the baseline and hyperoxygen pO_2_ values of individual patients were assessed using a two-tailed unpaired t-test. Significant differences between the group means of baseline and hyperoxygen pO_2_ values or between the results of the pre- and post-implant OxyChips were assessed using a two-tailed paired t-test. Pearson’s product-moment correlation coefficient (*r*) was used to assess two-way linear association between two continuous variables. Time-variation pO_2_ data sets from multiple measurements were fitted using a curve and estimated by nonlinear least squares using function nls in the R statistical package. The null hypothesis that the data on these patients belong to this curve was tested by chi-square. For all tests, a *P* value of ≤0.05 was considered statistically significant. Unless otherwise mentioned, the error bars represent standard error of the mean (SEM).

## Results

### Summary of pO_2_ Measurements

Twenty-three cancer patients with malignant tumors were enrolled and implanted with the OxyChip (**Table 1**). The tumor types included invasive ductal carcinoma (IDC) of the breast (patients 13–17, 19–21), squamous cell carcinoma (SCC) of the skin (patients 3,6,7,9–12,22,23), basal cell carcinoma (BCC, patients 5,24), sarcoma (patient 18), melanoma (patient 2,4), follicular thyroid carcinoma (patient 8). The Table also includes one patient with a non-malignant tumor (lipoma, patient 1). EPR measurements were carried out in all 24 patients over 71 visits, which included multiple visits in most patients—up to 7 visits over a period of up to 123 days post-implantation. Tumor pO_2_ values were successfully obtained in 16 patients over 46 visits, including measurement of response to hyperoxygen interventions in 43 visits. The first patient had a benign lipoma and therefore the pO_2_ data from this patient was not aggregated with that of the other 23 patients who had histologically documented malignant tumors.

**Table 1:** Patient information, OxyChip, and oxygen data.

Patient	Age	Sex	Clinical Diagnosis	Anatomical Location of Tumor	Treatment prior to Surgical Resection of Tumor (SOC)	OxyChip Implant Duration	Location of OxyChip in the Resected Tumor	Depth of OxyChip in Tumor	Post-implant Period at pO_2_ Scan (days)	Baseline pO_2_ Mean±SEM (mmHg)	Hyperox. pO_2_ Mean±SEM (mmHg)	Significance, P*
1	51	F	Lipoma	Upper left back, subcutaneous	None	5 days	Not within tumor; within superficial fascia of subcutaneous mass	< 10 mm	2	33.8±2.7	44.6±7.8	0.2369
2	69	F	Melanoma	Left anterior tibia, skin	None	4 days	Within tumor	3 mm	3	3.5±0.1	7.1±0.3	0.0000
3	61	M	SCC skin	Left nasal dorsum, skin	None	32 days	Within tumor	3 mm	8	1.4±0.5	1.5±0.5	0.6452
									32	0.6±0.1	6.7±2.1	0.0991
4	77	M	Melanoma	Scalp, skin	None	5 days	Within tumor	5–10 mm	5	9.3±0.5	5.6±0.7	0.001
5	69	M	BCC	Left temporal scalp, skin	None	33 days	Within tumor	2–3 mm	14	3.4±0.3	19.2±4.6	0.0255
									33	4.6±0.8	9.5±1.5	0.0065
6	63	M	SCC skin	Scalp, posterior superior, skin	None	Unknown	Not found, presumed lost prior to surgery due to rapidly progressive tumor necrosis	5 mm	23	NS	NM	
7	61	M	SCC skin	Right posterior triangle neck, subcutaneous mass	None	30 days	Outside of and adjacent to tumor within dermis	5 mm	3	33.7±0.1	96±2.9	0.0000
									9	15.9±1.4	50.3±2.7	0.0000
									21	21.1±2.2	NM	
									30	18.7±0.4	77.9±2.0	0.0000
8	56	M	FTC	Thyroid	None	47 days	Within tumor	25 mm	1,7,14	NS	NM	
9	72	F	SCC skin	Frontal scalp, left, skin	None	7 days	Within tumor	5-10 mm	1	6.0±0.2	60.3±10.4	0.0347
									4	7.9±0.4	108.5±5.1	0.0000
									6	9.6±1.9	127.1±9.4	0.0000
10	70	M	SCC skin	Infraorbital cheek, left, subcutaneous	None	25 days	Adjacent to tumor, but not within tumor; 4 mm from tumor margin	10 mm	1	47.5±1.6	67.0±2.9	0.0001
									16	10.5±0.2	16.5±0.7	0.0000
11	78	M	SCC skin	Right temporal scalp, skin	None	27 days	Within tumor	2 mm	8	13.3±0.7	62.4±5.7	0.0001
									22	1.8±1.1	4.4±1.6	0.1835
12	83	M	SCC skin	Right neck, level II lymph node	None	22 days	Within tumor	5 mm	1	10.4±0.5	6.1±0.0	0.0010
									14	2.0±0.7	NM	
13	42	F	IDC	Right breast	None	10 days	Within tumor	11 mm	3,7,10	NS	NM	
14	48	F	IDC	Left breast	None	13 days	Not within tumor, 1 mm from tumor edge	6 mm	1	13.3±1.4	64.3±6.2	0.0011
									4	16.4±2.4	88.0±12.8	0.0048
									6	24.5±1.1	64.5±3.1	0.0000
									7	17.6±3.0	56.6±6.7	0.0031
15	70	F	IDC	Left breast	Chemotherapy: paclitaxel / trasuzumab x 3 cycles	124 days	Uncertain relationship to pretreatment tumor	16 mm	9,15,31	NS	NM	
16	61	F	IDC	Left breast	Chemotherapy: carboplatin / docetaxel / trastuzumab / pertuzumab x 6 cycles	131 days	Uncertain relationship to pretreatment tumor	9.4 mm	16,34,99	NS	NM	
17	61	F	IDC	Left breast	Chemotherapy: dose dense adriamycin / cytoxan x 4 cycles	137 days	Uncertain relationship to pretreatment tumor	13 mm	6,20,62, 90,104	NS	NM	
18	23	M	Sarcoma	Right chest wall	Radiotherapy: 50 Gray	79 days	Within collagenous soft tissue skeletal muscle fascia outside of viable tumor at least 6 mm	18 mm	6,12,20, 27	NS	NM	
19	51	F	IDC	Right breast	Chemotherapy: carboplatin / docetaxel / trastuzumab / pertuzumab x 6 cycles	125 days	Uncertain relationship to pretreatment tumor. OxyChip not seen within small foci of residual tumor.	6–7 mm	24	12.4±0.5	20.5±1.3	0.0010
									45	16.2±1.3	NM	
									66	28.9±1.3	70.7±6.8	0.0002
									87	17.2±1.5	45.4±4.5	0.0006
									107	24.3±0.6	33.7±2.5	0.0079
20	55	F	IDC	Left axillary node	Chemotherapy: dose dense adriamycin / cytoxan x 1 cycle, transitioned to paclitaxel x 1 cycle	138 days	No residual tumor - uncertain relationship to pretreatment tumor	5–6 mm	13	36.3±3.7	144.6±19.7	0.0008
									30	23±1.2	56.3±2.8	0.0000
									58	18.4±1.8	37.1±3.5	0.0037
									86	4.4±0.5	10.9±4.5	0.2056
									99	21.9±1.0	23.7±2.6	0.4647
									112	15.7±1.5	14.9±3.8	0.8359
									124	7.6±0.4	11.5±1.3	0.0165
21	81	F	IDC	Right axillary node	None	20 days	Freely mobile within necrotic nodal tumor	10 mm	6	2.4±0.3	4.6±0.9	0.0329
									7	10.6±2.1	6.5±2.2	0.0080
									9	18.9±0.9	8.4±0.9	0.0200
									13	12.7±2.1	21.7±2.6	0.0011
									15	23.1±2.3	19.7±2.7	0.0786
22	65	M	SCC skin	Above manubrium, skin	None	42 days	Within lymph node, adjacent to nest of tumor	12.8 mm	28,30,35	NS	NM	
23	54	M	SCC HN	Level II LN, neck	None	11 days	Within tumor	10.5 mm	7	25.3±1.4	35.0±3.3	0.0188
									8	16.0±0.8	29.9±1.2	0.0000
									9	3.9±1.6	16.7±4.1	0.0290
24	53	M	BCC	Face, left, skin	None	Unknown	Not found, presumed lost at time of surgery	7 mm	5	73.1±4.9	89.0±10.4	0.3360
									21	69.9±18.6	80.7±19.8	0.4432

SCC, squamous cell carcinoma; BCC, basal cell carcinoma; FTC, follicular thyroid cancer; IDC, invasive ductal carcinoma; HN, head and neck; LN, lymph node; SOC, standard of care; NS, no signal; NM, not measured; *, two-tailed unpaired t-test between baseline and hyperoxygenation pO_2_ values.

### Patient Population, Treatment, and OxyChip Implantation

The median age of enrolled patients was 61 (range of 23–83). Women were 46% of the total cohort (n=24), and most patients had either an IDC of the breast (33%) or SCC of the skin (33%). Of the 24 patients implanted, definitive surgery alone was performed in 18 (median implant duration 21 days, range 4–42 days). Five patients received neoadjuvant chemotherapy followed by surgical resection (median implant duration 131 days, range 124–138 days); one received neoadjuvant radiotherapy followed by surgical resection (implant duration 79 days). The median time from OxyChip implantation to surgical removal for all patients was 29 days (range 4–138 days). Ultrasound image guidance was not used for placement in twelve patients (**Table 1**, patients 1–12), initially because the protocol did not incorporate imaging and later due to physician discretion relating to the superficiality and size of implanted malignancies.

### OxyChip Retrieval and Assessment of Implant Location in the Tumor

Pathological analysis was performed on the resected tumors to assess the OxyChip location. Of the 12 patients implanted without image guidance who received surgery alone, the OxyChip was found within the tumor in eight patients (**Table 1**, patients 2–5,8,9,11,12) and outside but adjacent to the tumor in three patients (patients 1,7,10). In one patient (patient 6), the OxyChip was found neither during EPR oximetry-measurement attempts nor on pathologic assessment. It was presumed that the OxyChip was inadvertently dislodged or fell out soon after implantation prior to initiation of oximetry measurements. Of the six patients implanted with image guidance who received surgery alone, the OxyChip was found within the tumor in three patients (patients 13,21,23) and outside but adjacent to the tumor in two patients (patients 14,22). In one patient (patient 24), the OxyChip was presumed to be lost at the time of surgery. Of the five patients implanted with image guidance who received chemotherapy, the OxyChips were within the tumor on initial placement (patients 15–17,19,20); however, determination of the location of the OxyChip relative to the tumor at pathological analysis was confounded by post-treatment effect (i.e., a decrease in the size of or complete resolution of the tumor due to a partial or complete response to therapy). In one patient (patient 18) who was implanted with image guidance and treated with neoadjuvant radiotherapy the OxyChip was assessed to be within the tumor on initial placement; however, it was found 6 mm outside viable tumor at pathologic analysis. Further evaluation of patient-reported outcomes, adverse events, and pathologic findings associated with OxyChip implantation have been published separately (51).

### OxyChip Measurements in the Short Term

**Figure 2:**
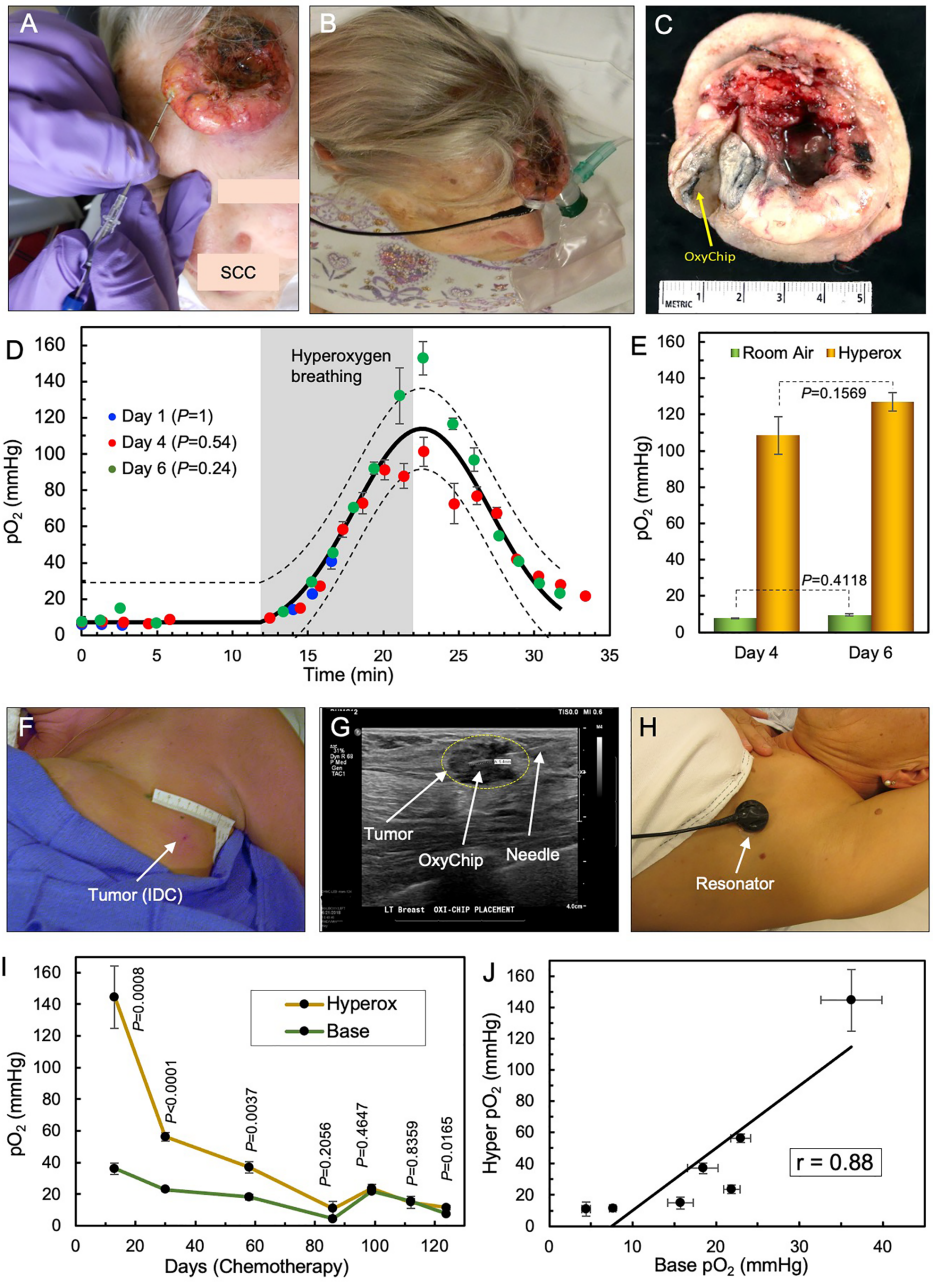
Repeated measurements of tumor pO_2_ using OxyChip. The reliability of the OxyChip for repeated measurements of tumor oxygen is demonstrated in two patients, an untreated SCC tumor in the short term and a breast tumor that was undergoing chemotherapy in the long term. **(A)** Implantation of the OxyChip in an untreated SCC on the left frontal scalp of a 72-year-old female (patient 9). **(B)** EPR measurement using a flexible surface-coil detector placed over the implant. **(C)** Surgically resected tumor (SOC therapy) showing the presence of the OxyChip in the tumor. **(D)** pO_2_ data (mean ± SEM) measured in three separate visits over a period of 6 days before tumor resection. The data were obtained during room-air breathing, hyperoxygen breathing and return to room-air breathing on days 1, 4, and 6 after implantation of the OxyChip. The solid and dotted black lines represent the mean curve and 95% CI, respectively, of all measurements, suggesting that the three sets of data are not significantly different from each other. Note that the gray representation of “hyperoxygen breathing”, as well as the time periods without oxygenation, are an average of the time for three sessions. The measurements on day 1 could not be continued beyond 5 minutes into hyperoxygenation due to technical reasons. **(E)** Mean (± SEM) baseline and estimated hyperoxygen pO_2_ values at 10 minutes for day 4 and 6 showing no significant difference between the baseline values or between the hyperoxygen values. **(F)** Implantation site of a left axillary node breast tumor (IDC) of a 55-year-old female (patient 20). EPR measurements occurred during seven visits while she underwent chemotherapy for > 4 months. **(G)** Ultrasound-guided implantation of the OxyChip in the tumor. The OxyChip has been deployed within the tumor, and the needle is being retracted. **(H)** EPR measurement using a flexible surface-coil detector placed over the tumor. **(I)** Changes in pO_2_ (baseline and response to hyperoxygenation; mean ± SEM) during the treatment period. The *P* values (unpaired t-test) represent significance of the hyperoxygenation values compared to corresponding baseline values. **(J)** Correlation between baseline and hyperoxygenation pO_2_ values (mean ± SEM) showing a strong positive correlation (Pearson’s correlation coefficient r=0.88).

The reliability of the OxyChip for short-term repeated measurements of pO_2_ is best illustrated in an untreated SCC on the left frontal scalp of a 72-year-old female (patient 9) in 3 visits over a period of 6 days. The selection of this cancer patient assumed that the implant would be within the tumor and that, in the short term (6 days) and without treatment, the baseline pO_2_ and its response to hyperoxygenation would not change significantly. **Figures 2A, B** shows the tumor on the scalp, OxyChip implantation, and detector placement during an EPR measurement. Microscopic examination of the resected tumor confirmed that the OxyChip was within the tumor, as evident on gross examination (**Figure 2C**). The pO_2_ data obtained during room-air breathing, hyperoxygen breathing (100% O_2_) and return to room-air breathing on days 1, 4, and 6, shown superimposed in **Figure 2D**, exhibited a reproducible pattern—both in trend and magnitude. Non-linear least-squares fitting of the time variation of pO_2_ data sets revealed that there were no significant differences between all three measurements. The mean baseline and estimated hyperoxygen pO_2_ values were similar (**Figure 2E**).

### OxyChip Measurements in the Long Term

The capability of the OxyChip for long-term monitoring of pO_2_ in tumors is best illustrated in a 55-year-old female with breast cancer (**Table 1**, patient 20). She had EPR measurements over 7 visits spanning a period of 124 days while undergoing dose-dense chemotherapy (doxorubicin/cyclophosphamide followed by paclitaxel). **Figures 2F–H** shows the site of implantation in a left axillary node, OxyChip implantation using ultrasound guidance, and EPR measurement using the flexible surface-coil detector (53). Repeated measurements of tumor pO_2_ and response to hyperoxygen breathing during the treatment period showed a progressive decline of baseline pO_2_ for about 3 months (36.3 ± 3.7 mmHg on day 13 to 4.4 ± 0.5 mmHg on day 86) followed by an increase for a brief period (15.7 ± 1.5 on day 112) and eventually dropping to 7.6 ± 0.4 mmHg on day 124 (**Figure 2I**). Hyperoxygenation exhibited an increase in pO_2_ and followed a similar trend suggesting a strong positive correlation (*r*=0.88) between baseline and hyperoxygenation pO_2_ values (**Figure 2J**).

### Stability of the OxyChip and Oxygen Sensitivity in Tumors

**Figure 3:**
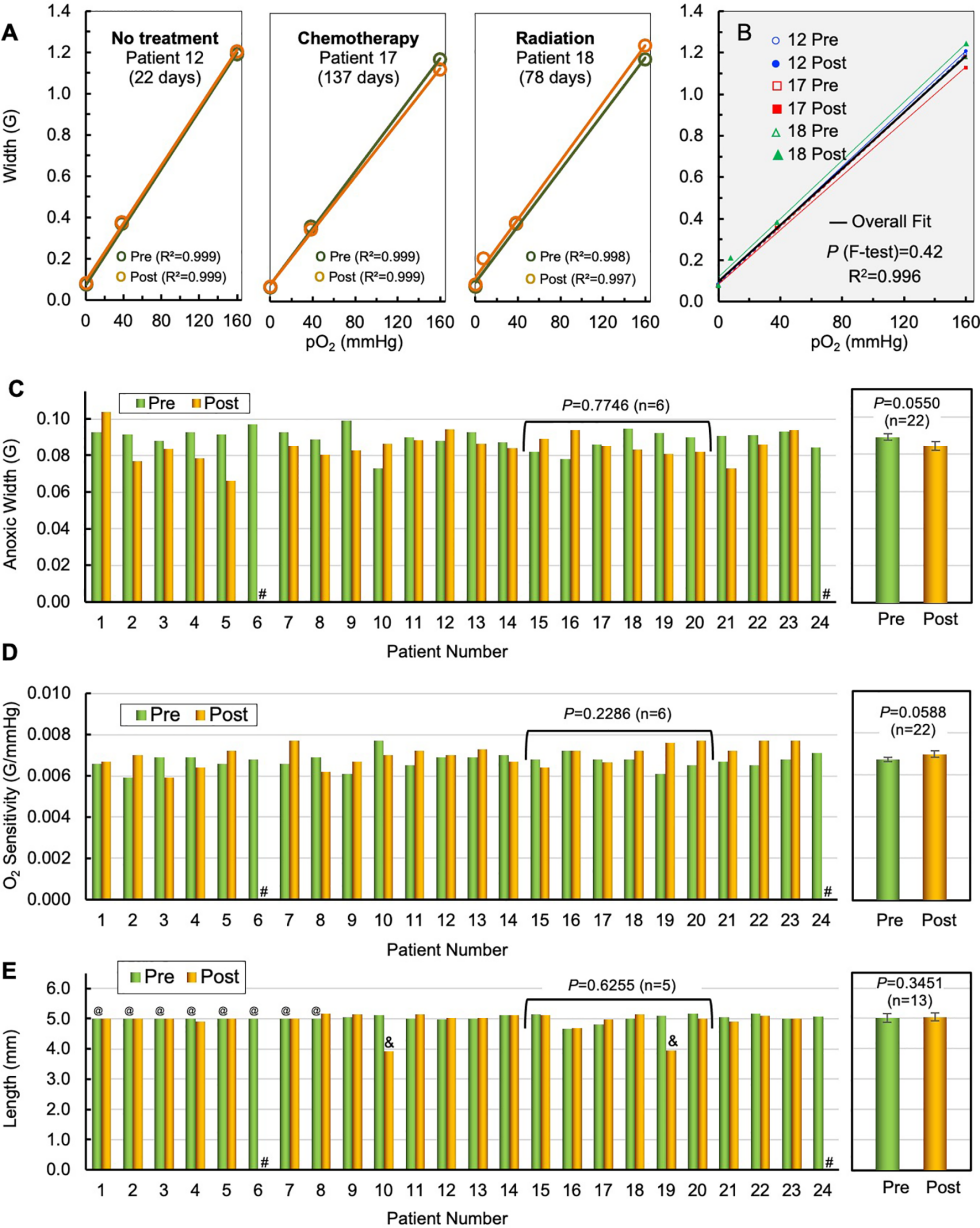
Stability of OxyChip implant and function in tumors—during residency and treatment. The stability of the OxyChip for long-term monitoring of pO_2_ in a variety of human tumors, implant-periods, and treatments was evaluated by checking pre/post calibration of their oxygen sensitivity and structural integrity (morphology) of the implant. Representative calibration data include OxyChips removed after: **(A)** 22 days in an untreated SCC (patient 12); 137 days in a breast tumor (IDC) treated with chemotherapy (patient 17); and 78 days in a sarcoma (patient 18) treated with radiation. There were no apparent changes in the calibrations including linearity between pre- and explanted OxyChips. **(B)** F-test and overall fit of all three pre- and post-implant OxyChips do not show any statistically significant differences in their calibration. **(C)** EPR spectral width of pre- and post-implant OxyChips under anoxic condition (Anoxic Width). ^#^Lost OxyChip. There was no overall significant difference in the anoxic width between the pre- and explanted OxyChips (22 OxyChips, mean ± SEM, paired t-test, *P*=0.0550). There were also no significant differences among the OxyChips from patients 15–20 that underwent chemo- or radiation therapy during implant (paired t-test, *P*=0.7746; n=6). **(D)** Pre- and post-implant oxygen sensitivity of each OxyChip. ^#^Lost OxyChip. There was no overall significant difference in the oxygen sensitivity between the pre- and explanted OxyChips (22 OxyChips, mean ± SEM, paired t-test, *P*=0.0588). There were also no significant differences among the oxygen sensitivity of the OxyChips from patients 15–20 that underwent chemo- or radiation therapy during implant (paired t-test, *P*=0.2286; n=6). **(E)** Pre- and post-implant length of OxyChips. There were no overall significant differences between the pre- and post-implant OxyChips (13 OxyChips, mean ± SEM, paired t-test, *P*=0.3452). There were also no significant differences among the OxyChips from patients 15–20 (patient 19 excluded) that underwent chemo- or radiation therapy during implant (paired t-test, *P*=0.6255; n=5). Key: ^#^Lost OxyChip; ^@^Made to 5-mm length, but not measured before implantation; ^&^Possibly cut during recovery.

The stability of the OxyChip for long-term monitoring of pO_2_ in a variety of human tumors and implant periods was determined by comparing the pre- and post-implant oxygen sensitivity and structural integrity data for each OxyChip. Data from all 22 OxyChips that were recovered after removal en bloc in SOC surgery were used in this analysis. The recovered OxyChips were sterilized before examination. They were then physically measured for length and microscopically examined for any surface irregularity different from what was recorded for the control, i.e., the same OxyChip examined pre-implant. The stability of the oxygen sensitivity of the recovered OxyChips was determined by recalibration of each explanted OxyChip and comparing to its pre-implantation data. **Figure 3A** shows the pre- and post-implant calibration curves for three specific cases, namely an OxyChip from (i) an untreated SCC tumor implanted for 22 days, (ii) a breast tumor that was treated with chemotherapy while the implant was in the tumor for 137 days, and (iii) a sarcoma that was treated with radiotherapy while the implant was in the tumor 78 days. **Figure 3B** superimposes all six pre- and post-implant calibration curves onto the same graph, illustrating the similarity across OxyChips as well as pre-and post-implantation. The results showed no significant differences between the six pre- and post-implantation calibrations for the three OxyChips (*P*=0.42). In all three cases, there were no differences between the pre- and post-implant OxyChip calibrations suggesting that none of the factors—residency in untreated tumors, chemotherapy, or radiation therapy—affected the oxygen sensitivity as assessed by re-calibrating the OxyChip. The pre- and post-implant values of anoxic width and oxygen sensitivity, both of which are critical parameters of oxygen calibration, are shown for all 22 recovered OxyChips in **Figures 3C, D**. The mean values of anoxic width and oxygen sensitivity of the OxyChips were not significantly different when compared to their pre-implant values (*P*=0.0550 and *P*=0.0588, respectively; n=22). A similar analysis in a subset of cancer patients who received neoadjuvant chemotherapy or radiation therapy during the implant duration did not show any significant difference between the pre- and post-implant values of anoxic width (*P*=0.7746; n=6) or oxygen sensitivity (*P*=0.2286; n=6). Structural integrity of the OxyChips was based primarily on a comparison of pre- and post-implant length and also on the microscopic examination. Of the 22 OxyChips recovered from the resected tumor, 2 were found to be shorter in length compared to pre-implant values (3.9 vs 5.1 mm from patient 10 and 4.0 versus 5.0 mm from patient 19), apparently due to being cut during the bread-loafing procedure used to find the OxyChip in the tissue specimen. Seven OxyChips (**Table 1**, patients 1–7), although fabricated to 5-mm length, were not measured before implantation as indicated in **Figure 3E**.

The mean value of the length of the recovered OxyChips (5.04 ± 0.13 mm; n=13), excluding the two cut-off and seven not pre-measured OxyChips, did not change significantly from the pre-implant value (5.01 ± 0.13; n=13; *P*=0.3451). Further, a similar analysis in a subset of cancer patients who received neoadjuvant chemotherapy or radiation during the implant duration did not show any significant difference between the pre- and post-implant values of the OxyChip length (*P*=0.6255; n=5). This subset, although small, was considered to be an important confirmation because they were implanted the longest and were exposed *in situ* to the SOC cancer treatments. Of note, the size of the OxyChip (whole or cut-off) does not affect the oxygen sensitivity; however, all pieces (if any) were used in the calibration tests reported in **Figures 3A–D**.

### Baseline Tumor pO_2_ and Response to Hyperoxygen Breathing

**Figure 4:**
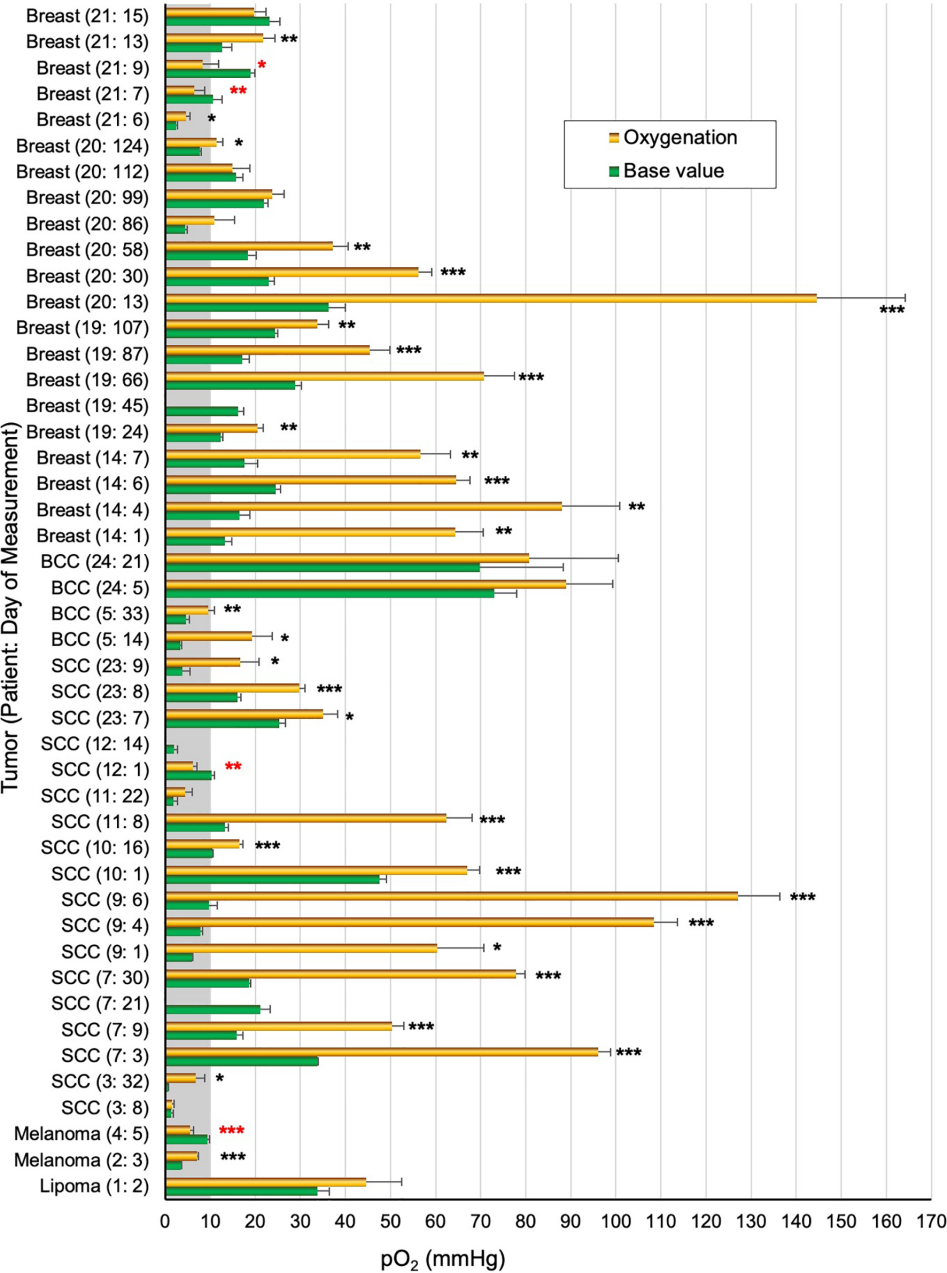
Tumor pO_2_ values in patients breathing room air and hyperoxygen gas. The pO_2_ values (mean ± SEM) obtained before initiation of hyperoxygenation (Base value) and after hyperoxygenation (Hyperoxygenation) in a total of 46 measurements from 16 patients. For each measurement the tumor type, patient number, and measurement day relative to initial implantation are noted, i.e. “Breast (21:5)” indicates that patient 21 had a breast malignancy and this measurement occurred on day 5 after OxyChip implantation. Multiple measurements from the same patient are thus indicated by different days relative to OxyChip implantation. Statistical significance data (unpaired t-test) between base and hyperoxygenation pO_2_ values for each patient/measurement are grouped as *P≤0.05; **P<0.01; ***P<0.001 (actual *P* values are in **Table 1**). A red-colored * denotes significantly negative response to hyperoxygenation.

**Figure 4** provides a summary of pO_2_ values measured in 16 patients during a total of 46 visits. The number of measurements for each patient ranged from 1–7. Multiple measurements from the same patient, including the number of days that the OxyChip was implanted, are indicated numerically in the format (patient number: day of measurement) in **Figure 4**. Of the 46 patient-sessions measured during room-air breathing, 43 patient-sessions, including all 16 patients, underwent further measurements during hyperoxygen breathing. Statistical significance (*P* values) for comparing the base and hyperoxygenation pO_2_ values of the 43 patient-sessions are indicated in **Table 1**.

Overall, excluding patient 1, the baseline pO_2_ data from the malignant tumors ranged from 0.6 to 73.1 mmHg (mean 17.2 ± 2.3, median 15.7 mmHg, n=45), while hyperoxygen values ranged from 1.5 to 144.6 mmHg (mean 42.4 ± 5.7, median 31.8 mmHg, n=42). Of the 45 baseline values, 15 (33%, 10 patients) were below 10 mmHg and 10 (22%, 8 patients) were below 5 mmHg. Hyperoxygen intervention showed a significantly higher pO_2_ (positive response) in 30 measurements (71%, 13 patients), while a significantly lower pO_2_ (negative response) was observed in 4 measurements (10%, 3 patients). Eight measurements (19%, 5 patients) did not show any significant response to hyperoxygen breathing. It should be noted that in four patients (**Table 1**, patients 3,11,20,21) the hyperoxygen responses were mixed (positive to non-response) on repeated measurements.

### Mitigation of Tumor Hypoxia by Breathing Hyperoxygenated Gas

**Figure 5:**
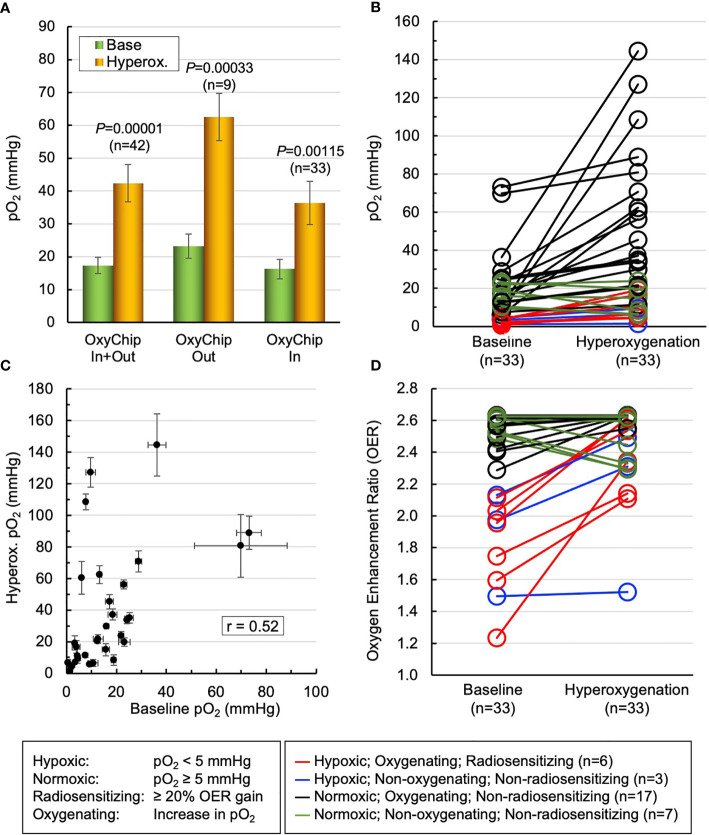
Mitigation of tumor hypoxia for therapeutic enhancement. The pO_2_ data from 33 measurements in 12 patients (patients 2–5,9,11,12,19–21,23,24), wherein the OxyChips were found inside the tumor in the resected specimen or placed in the tumor during ultrasound-guided implantation but unable to ascertain their location after neoadjuvant treatment, were used to identify the population of hypoxic tumors and responders to hyperoxygen intervention. **(A)** The first pair of bars give the values for the measurements in all malignant tumors. The second and third pairs show the measurements for the malignant tumors in which the OxyChip was or was not found to be in the tumors at the time of resection. Within the third selected group of patients the mean of base pO_2_ values was 16.3 ± 2.9 mmHg, while that of hyperoxygen pO_2_ values was 36.4 ± 6.6 mmHg (*P*=0.00115). **(B)** A pair-wise representation of the base and hyperoxygen pO_2_ values in the 33 measurements in which the OxyChip was in the tumor. **(C)** Correlation between the baseline pO_2_ and its response to hyperoxygenation in the measurements in these selected tumors, exhibiting a moderate correlation (Pearson’s correlation coefficient *r*=0.52). **(D)** Level of radio-sensitization, in terms of oxygen enhancement ratio (OER), by hyperoxygenation. Nine measurements showed severely hypoxic (pO_2_ < 5 mmHg) tumors in which six could be sensitized to radiation (i.e., showed ≥ 20% OER gain). Twenty-four measurements had pO_2_ ≥ 5 mmHg and, irrespective of whether they responded or not, hyperoxygenation probably would not have had a beneficial radio-sensitizing effect in these tumors at the times measured.

The overarching goals of EPR oximetry using the OxyChip are to identify tumor hypoxia and stratify patients as responders or non-responders to hypoxia-mitigation interventions to optimize oncologic outcomes (e.g., *via* radiotherapy). To study the potential for clinically relevant stratification of tumors to determine the efficacy of hyperoxic interventions, and to examine the impact of oxygenation interventions to increase tumor oxygen in clinical relevant ways, we used the data on pO_2_ from 33 measurements in the 12 patients (**Table 1**, patients 2–5,9,11,12,19–21,23,24), in which the OxyChips were either found within the tumor at resection or known to have been placed into the tumor during implantation *via* ultrasound imaging (because these tumors shrank very significantly in response to treatment it was not feasible to determine their position within the tumor during measurements by post-resection evaluation). Within this selected group of patients, the mean of the base pO_2_ values was 16.3 ± 2.9 mmHg (median 12.4 mmHg, range 0.6 to 73.1 mmHg), while the mean of hyperoxygen values was 36.4 ± 6.6 mmHg (median 20.5 mmHg, range 1.5–144.6 mmHg), These data likely represent a more accurate measure of tumor oxygen status and response to hyperoxygen intervention in the types of tumors for which clinical intervention is most likely to be relevant, as compared to the entire cohort (**Figure 5A**). A pair-wise representation of base and hyperoxygen values from 33 measurements (in the 12 patients) where the OxyChip was in the tumor is shown in **Figure 5B**. We further tested to see whether a correlation existed between the base pO_2_ and its response to hyperoxygenation in these tumors. The data, as shown in **Figure 5C**, showed a Pearson’s correlation coefficient r=0.52 suggesting a moderately positive correlation between the base pO_2_ and response to hyperoxygenation.

Since it is known that the radiobiological response of cancers is critically dependent on tumor oxygenation, especially hypoxic pO_2_ values, we converted the oxygenation data into the oxygen enhancement ratio (OER) as modeled and reported by Grimes and Partridge (56). **Figure 5D** depicts a view of the level of radio-sensitization, in terms of OER, by hyperoxygenation. The results indicated that in about 27% of the measurements (9 out of 33) the tumors were severely hypoxic (pO_2_ < 5 mmHg) and 67% (6 out of 9) of them could be sensitized to radiation with ≥20% OER gain. Further, about 42% of the measurements (14 out of 33) were radiobiologically hypoxic (pO_2_ < 10 mmHg) and 64% (9 out of 14) of the hypoxic tumors could be sensitized to radiation with ≥10% OER gain. Twenty-four measurements (73%) showed the tumors were normoxic (pO_2_ ≥ 5 mmHg) and, consequently, irrespective of whether they responded or not to hyperoxygenation, there may not have been an appreciable radiosensitiziation impact at the times measured. Overall, these results emphasize the need to measure pO_2_ in each tumor at the time of treatment in order to optimize hypoxia-mitigation strategies.

## Discussion

The results of the present study establish, for the first time, that tumor oxygen levels can be measured in cancer patients repeatedly using EPR oximetry with the OxyChip to obtain both initial baseline values and values after interventions designed to increase tumor oxygenation. Importantly, these results also demonstrate the capacity to measure these values successfully over long periods of time. The oxygen data obtained from a small cohort of patients showed considerable variations among tumors as well as in the same tumor, with or without cancer-directed therapy, as a function of time. Clinically significant hypoxia was observed in a subset of tumors with varying levels of response to hypoxia-mitigation by breathing oxygen-enriched gas. However, despite the significant heterogeneity of tumor pO_2_ we observed statistically significant increases in tumor oxygen following administration of hyperoxygenated gas across various types of tumors and patients. This finding suggests that the OxyChip has the potential to measure subtle changes of tumor oxygen before and during cancer treatment and as such paves the way for using EPR oximetry in the clinical setting for cancer prognosis and treatment planning. Overall, the results underscore the importance of individualized measurements of tumor oxygen levels, both at baseline and in response to hyperoxygenation interventions designed to optimize therapeutic outcomes.

The objectives of this first-in-human device study were to establish the safety of OxyChip implantation and subsequent EPR oximetry in human tumors, as well as to establish the feasibility of using the OxyChip for repeated measurements of tumor oxygen to obtain clinically useful data. We have recently reported on the safety of the OxyChip after implantation into human tumors (51). The results showed that OxyChip implantation followed by EPR oximetry was safe and feasible without any significant clinical adverse effects in all 24 patients studied. Both the implantation procedure and the process of EPR oximetry in the clinic were well tolerated by the patients. Histopathologic findings revealed no clinically significant pathology, indicating that the tissue reaction to the OxyChip was well within expectations for an implanted device.

Previously, we have established the OxyChip to be a robust, stable, and reliable sensor for repeated measurements of pO_2_ for up to one year using a rat model (36). In the present study, the OxyChips were implanted in a variety of human tumors (n=24) with implantation periods (4–138 days) during no treatment (n=16), chemotherapy (n=5), and radiotherapy (n=1). The OxyChips that have been successfully recovered in 22 patients appeared to have been unaffected—in terms of structural integrity and oxygen sensitivity—by the variable periods of residency in the tumors. Importantly, the OxyChips appeared to be unaffected by chemotherapy in all patients undergoing chemotherapy (five patients, 124–138 days) and radiotherapy (in one patient, 78 days) suggesting that they could be reliably used to monitor changes in pO_2_ during or post-treatment. It should be noted that although ionizing radiation such as X-rays theoretically may induce some transient or permanent damage to the OxyChip, radiation doses of up to 80 Gy did not impair its oxygen-sensing ability in rat muscle (36). Furthermore, oxygen-sensing is an intrinsic property of the OxyChip; its size or shape (linear, bent, or physically damaged) does not affect its response to oxygen. This is particularly useful for implanting OxyChips of any shape and size, as may be needed for a given study (57, 58).

Successful pO_2_ measurements were made in 16 of the 24 patients measured. Except in patient 6, where the OxyChip inadvertently dislodged or fell out soon after implantation prior to initiation of oximetry measurements, in all other cases the OxyChips were in the tumor or in tissue nearby at the time of EPR measurements. The inability to detect a signal from the other 7 patients may have been due to the implant depth from the skin surface of the tumor or from tissue mobility, e.g., in breast tissue. From the gross estimate of the implant depth in the resected tumors in the pathology lab and/or from the ultrasound-guided placement of the OxyChips presented in **Table 1** (‘Depth of OxyChip in Tumor’), it appears that the implants at depths of approximately >10 mm were not detectable (**Supp. Figure 4**). Of note, as four of these were breast tumors, we suspect that the nature of the mammary tissue may play a role in the attenuation of RF power penetration. Nevertheless, the data from the present study suggest that the measurable depth limit is about 10 mm in human tumors using the current procedures, OxyChip configuration, and EPR instrument.

The data presented here, although in a relatively small cohort of patients, highlight the potential impact of insight into tumor hypoxia both prior to and during hyperoxygenation interventions. Importantly, EPR oximetry allows evaluation of the pO_2_ surrounding the OxyChip. In human tumors the pO_2_ can change, in an unpredictable fashion, with administration of oncologic therapies over prolonged periods of time. For example, in patient 20 (IDC of the left breast, with implantation into an axillary node) while the malignancy appeared initially to be normoxic, with progressive administration of chemotherapy over four months it appeared to become sequentially more hypoxic, eventually reaching a low value of pO_2_ < 5 mmHg prior to rebounding slightly. However, in patient 19 (also with an IDC of the left breast, implantation into the primary malignancy) both before and after administration of chemotherapy over four months, the tumor appeared to never become hypoxic. This variation in baseline pO_2_ also occurred for individual tumors without any oncologic intervention (for example, patient 11 with a SCC of the skin who received surgery alone, whose cancer was not hypoxic with a pO_2_ of 13.3 mmHg on day 8, but hypoxic on day 22 with a pO_2_ of 1.8 mmHg). These data highlight the need to understand tumor hypoxia at the point of interest, i.e., immediately prior to interventions designed to impact hypoxia.

Increased tumor oxygenation in and of itself is not necessarily clinically useful, and in an attempt to understand the impact of hyperoxygenation, an exploratory analysis on a subset of implanted cancers in which the OxyChip was clearly intra-tumoral was performed (**Figure 5**). Oxygenated tumors have been reported to respond better to radiotherapy by a factor 2.5–3 relative to anoxic tumors (59–61). Based on the hypothesis that the radiation-induced cell killing is due to permanently fixing the radical-mediated DNA damage by molecular oxygen and thus making the DNA damage irreparable, Grimes and Partridge (56) proposed a model for expressing the oxygen enhancement of cell killing and validated it using reported data on experimental oxygen curves (59–61). The model calculates oxygen enhancement ratio (OER), which is defined as a fold-increase in radiosensitivity by tumor oxygen relative to anoxic tumor. This analysis reveals the utility of understanding baseline oxygen and the response to hyperoxygenation *in situ* at the time of intervention.

In our subset study of measurements made in malignant tumors, only a small number of measurements (9 out of 33 in 7 patients) demonstrated severe hypoxia at baseline, and of these, only 6 (in 5 patients) responded to hyperoxygenation with a gain of ≥20% oxygen enhancement ratio (OER). This set of patients, as their tumors were found to be both hypoxic and responsive, would be prime candidates for hyperoxygenation during radiotherapy. In contrast, 24 of the measurements in malignant tissue revealed baseline pO_2_ > 5 mmHg and, although increase in pO_2_ was evident after hyperoxygenation in 17 measurements, if there were no areas that were more hypoxic, the radiobiological impact is expected to be modest or negligible. Although this analysis is clearly speculative, it may serve as a useful framework for clinical-trial design and interpretation. In particular it would be very useful to carry out clinical trials in which hyperoxygenation strategies are combined with serial measurements of tumor oxygen, using clinical outcomes to determine the circumstances in which hyperoxygenation can improve the efficacy of tumor therapy.

The stable and linear calibration of the OxyChip, combined with its fast time-response can enable clinically feasible monitoring of dynamic changes of tumor pO_2_. As demonstrated here, measurements can be obtained both prior to and during hyperoxygenation over many months. The promise of EPR oximetry to make repeated pO_2_ measurements in human tumors under clinically applicable conditions could have a significant impact on routine clinical decision-making processes by making previously unavailable information more easily accessible. Information regarding the presence of tumor hypoxia before the treatment can help to identify those patients who can (and cannot) benefit from a proposed therapeutic procedure. Furthermore, knowledge about the changes in tumor oxygenation induced by a given hyperoxic protocol, may lead to individualization of hypoxia-mitigation strategies. In so doing, EPR oximetry may be a useful component of personalized precision medicine with respect to hypoxia interventions.

In the process of establishing the feasibility of the technology, we have also identified some potential limitations, which need to be addressed in order to improve the applicability of this technology to a wide range of cancers. In this study, the EPR oximetry uses a stable implantable sensor, the OxyChip, which is useful for repeated, longitudinal monitoring of oxygen levels during treatment. The method is minimally invasive in that it requires one-time implantation of the sensor(s) and, at present, its surgical removal; however, subsequent measurements are made noninvasively and repeatedly over the long-term while it remains implanted. The potential limitation of providing data from one site and not providing information on oxygen distribution in the entire tissue may be mitigated using multiple implants to assess heterogeneity of oxygen (57, 62), or by combining EPR oximetry with volumetric methods of oxygen assessment (63–65). The operational frequency, approximately 1,150 MHz, of the current EPR scanner limits the use of the OxyChip to superficial tumors; however, this limitation can be overcome using implantable resonators containing OxyChip-like sensors (66, 67) or low-frequency and pulse EPR methods (68–71). Potential improvements in the future could also include the use of other clinical imaging modalities in addition to ultrasound, e.g., CT or PET, to improve both the precision of implantation and visualization of the implant in the tumor during treatment and pO_2_ measurements (72). Furthermore, multiple smaller implants to assess tumor oxygen heterogeneity, as well as the ability to measure deeper tumors, are needed to expand the utility of EPR oximetry for a wider range of tumors and treatment. We are committed to continue our efforts to address these improvements.

## Conclusion

The data from this study support the feasibility of using EPR oximetry to identify the presence of hypoxia and to identify the potential for hyperoxic therapy to improve tumor oxygen in each patient. This report of the first-in-human study of EPR oximetry using the OxyChip demonstrated variable levels of (i) tumor oxygen among patients, (ii) clinically significant hypoxia, and (iii) response to hyperoxygen. These data highlight the need for individualized assessment of tumor oxygenation in the context of planned hyperoxygenation intervention to optimize clinical outcomes.

## Data Availability Statement

The original contributions presented in the study are included in the article/**Supplementary Material**. Further inquiries can be directed to the corresponding author.

## Ethics Statement

The studies involving human participants were reviewed and approved by The study protocol (IRB Study 00028499) was approved by the Institutional Review Board (IRB) at Dartmouth College and Dartmouth-Hitchcock Medical Center (DHMC). The patients/participants provided their written informed consent to participate in this study.

## Author Contributions

Study conception and design: PS, BW, EC, LJ, HS, and PK. Clinical protocol development: PS, BW, EC, LJ, PF, and PK. Protocol maintenance: PS, LJ, VW, AF, KH, and PK. OxyChip fabrication and characterization: MK, HH, DT, and AP. Clinical coordination: PS, LJ, VW, and KH. OxyChip implantation and clinical imaging: PS, EC, LJ, DP, RZ, and RD-A. Surgical procedure: JAP, BG, RB, and KR. Pathological analysis and interpretation: JRP and PS. Clinical EPR measurement: BW, WS, MK, VW, and KH. EPR data acquisition, software, fitting, and analysis: BW, WS, MK, and PK. Engineering support: BW, WS, MK, and SP. Statistical analysis: ED and PK. Data interpretation and discussion: PS, BW, AF, HS, and PK. Manuscript planning, writing and illustration: PS, MK, and PK. Administrative support: VW, KH, and RM. Study supervision: PS and PK. All authors contributed to the article and approved the submitted version.

## Conflict of Interest

PK has multiple patents issued for OxyChip; but declares no competing interest. AF and HS are co-owners of Clin-EPR, a company that sells EPR spectrometers for research use in human subjects.

The remaining authors declare that the research was conducted in the absence of any commercial or financial relationships that could be construed as a potential conflict of interest.

## Publisher’s Note

All claims expressed in this article are solely those of the authors and do not necessarily represent those of their affiliated organizations, or those of the publisher, the editors and the reviewers. Any product that may be evaluated in this article, or claim that may be made by its manufacturer, is not guaranteed or endorsed by the publisher.
