# Supplementary material for: First-In-Human Study in Cancer Patients Establishing the Feasibility of Oxygen Measurements in Tumors Using Electron Paramagnetic Resonance With the OxyChip
Source: Front Oncol. 2021 Oct 1;11:743256. doi: 10.3389/fonc.2021.743256 (PMC8517507; doi:10.3389/fonc.2021.743256)
Supplement: Supplementary file 1 [file DataSheet_1.docx]

**First-in-human study in cancer patients establishing the feasibility of oxygen measurements in tumors using electron paramagnetic resonance with the OxyChip**

Philip E. Schaner^1^, Benjamin B. Williams^1,4^, Eunice Y. Chen^2^, Jason R. Pettus^3^, Wilson A. Schreiber^4^, Maciej M. Kmiec^4^, Lesley A. Jarvis^1^, David A. Pastel^4^, Rebecca A. Zuurbier^4^, Roberta M. DiFlorio-Alexander^4^, Joseph A. Paydarfar^2^, Benoit J. Gosselin^2^, Richard J. Barth^2^, Kari M. Rosenkranz^2^, Sergey V. Petryakov^4^, Huagang Hou^4^, Dan Tse^4^, Alexandre Pletnev^5^, Ann Barry Flood^4^, Victoria A. Wood^4^, Kendra A. Hebert^4^, Robyn E. Mosher^4^, Eugene Demidenko^6^, Harold M. Swartz^4^, Periannan Kuppusamy^1,4,5^

Norris Cotton Cancer Center, Geisel School of Medicine, Dartmouth College, Hanover, New Hampshire, USA. Dartmouth-Hitchcock Medical Center, Lebanon, New Hampshire, USA. Departments of Medicine^1^, Surgery^2^, Pathology^3^, Radiology^4^, Chemistry^5^, and Biomedical Data Science^6^, Dartmouth College, Hanover, New Hampshire, USA

# Supplementary Data

**
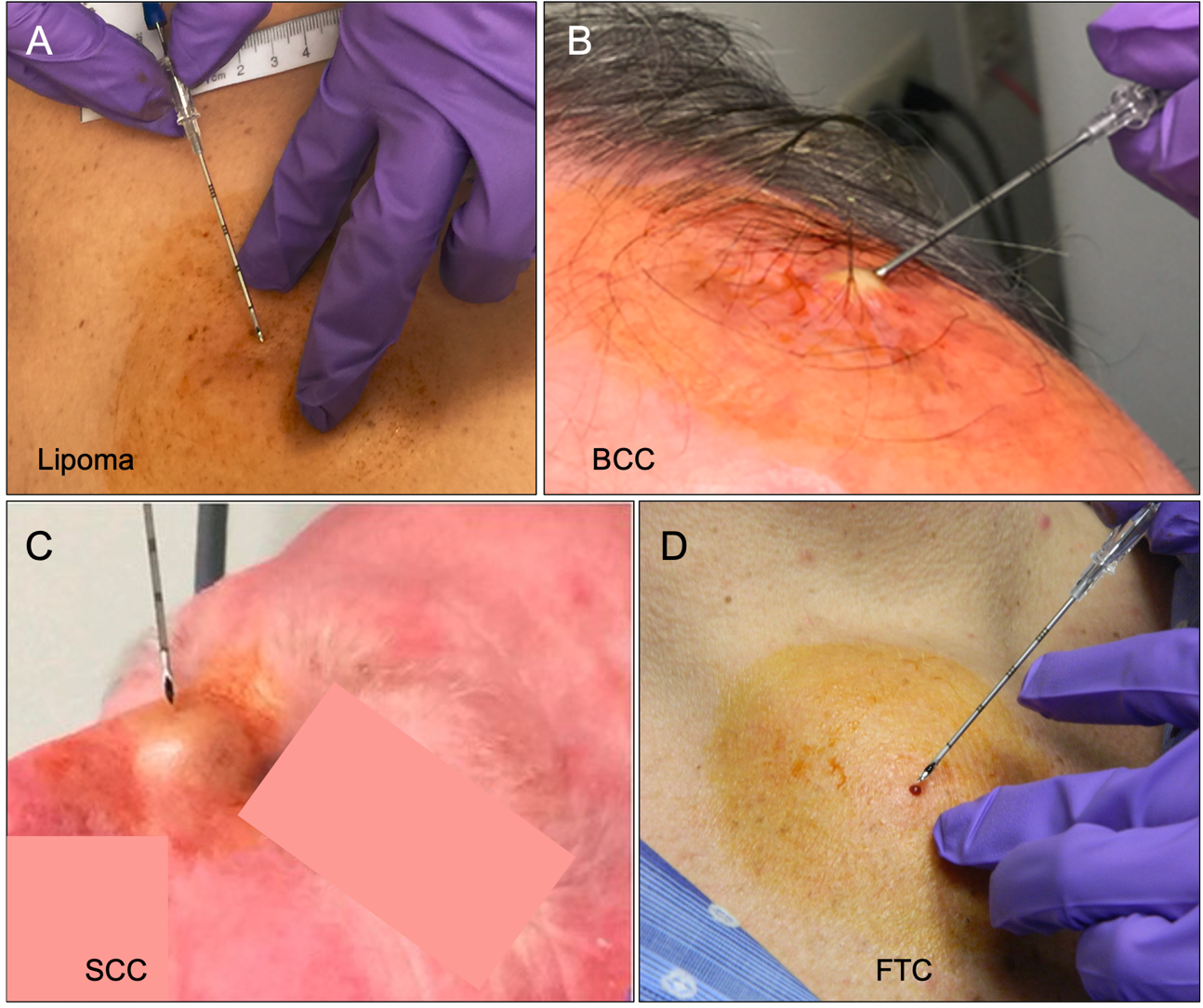
**

**Supp. Fig. 1 OxyChip implantation.** The OxyChip placed in the tip of an 18G brachytherapy needle was inserted into the tumor under local anesthesia (1% lidocaine) and deployed under sterile conditions. Ultrasound guidance was used wherever necessary; however, it was not in the examples shown here. Representative implantations are shown for (**A**) subcutaneous lipoma on upper left back (**Table 1**, patient 1); (**B**) Basal cell carcinoma (BCC) on left temporal scalp (**Table 1**, patient 5); (**C**) Squamous cell carcinoma (SCC) on left nasal dorsum (**Table 1**, patient 3); and (**D**) follicular thyroid cancer (FTC) (**Table 1**, patient 8).

**
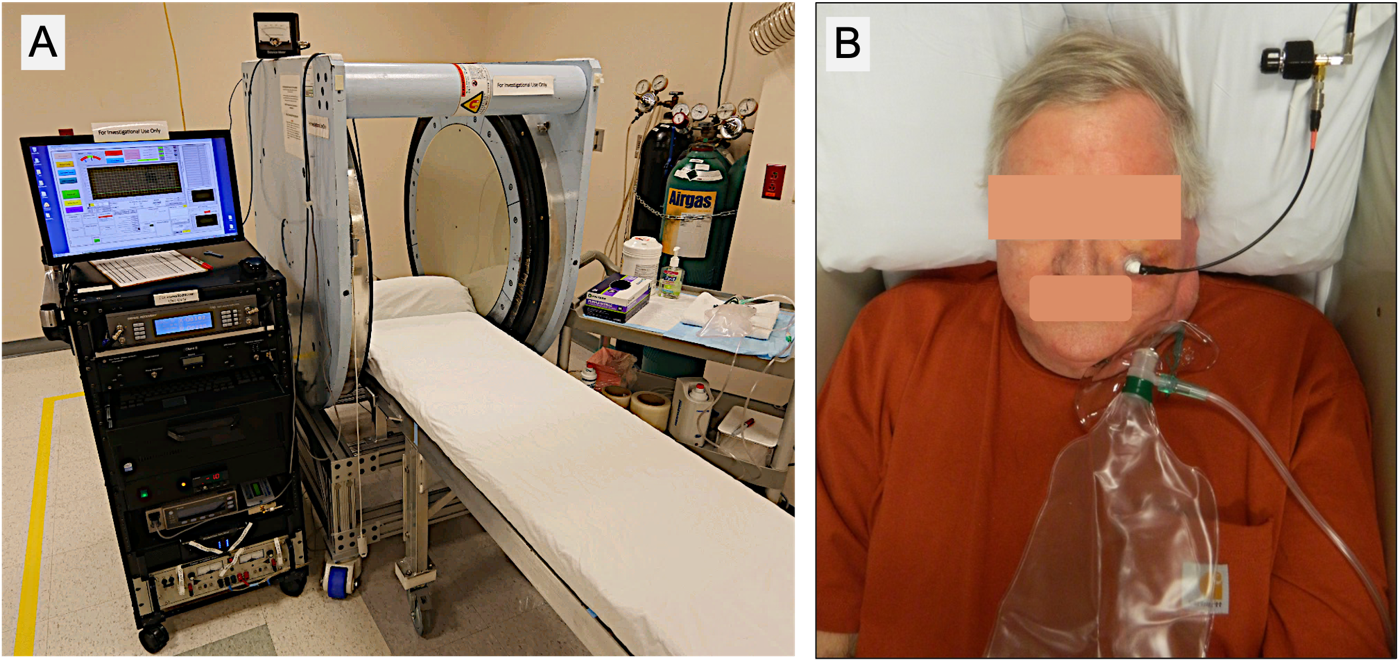
**

**Supp. Fig. 2 EPR measurements.** (**A**) A custom-built EPR scanner working at ~1.1 GHz (0.041T) was used for in vivo EPR oximetry. Patients were placed on a gurney and easily moved in/out of the magnet. (**B**) A patient (**Table 1**,10) in the magnet undergoing EPR scan using a flexible surface-coil detector securely placed over the tumor. Also shown is a non-rebreathing face mask for administering hyperoxygenated gas.

**
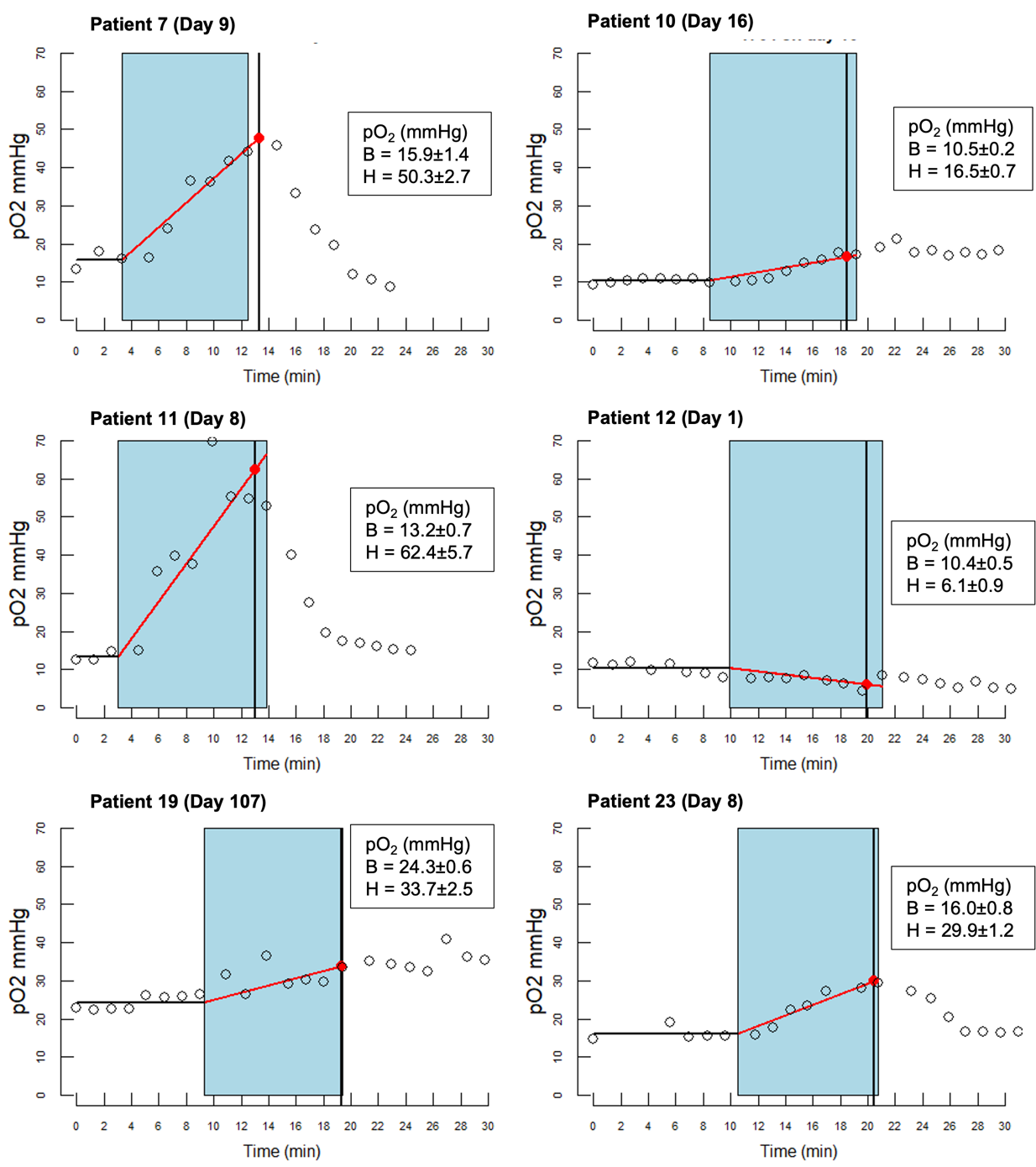
**

**Supp.** **Fig. 3 Estimation of baseline and hyperoxygenation pO_2_ values**. Representative examples of estimation of baseline and hyperoxygen pO_2_ values by fitting are shown. The baseline pO_2_ represents the mean±SEM of the pO_2_ values obtained while the patient breathed room air, before being switched to hyperoxygen breathing. The hyperoxygenation pO_2_ value is obtained as an estimate from a linear fitting of the pO_2_ values during hyperoxygen breathing. This value represents the pO_2_ (±SEM) at the end of 10 min after switching to hyperoxygen breathing, regardless of actual duration of hyperoxygenation.

**
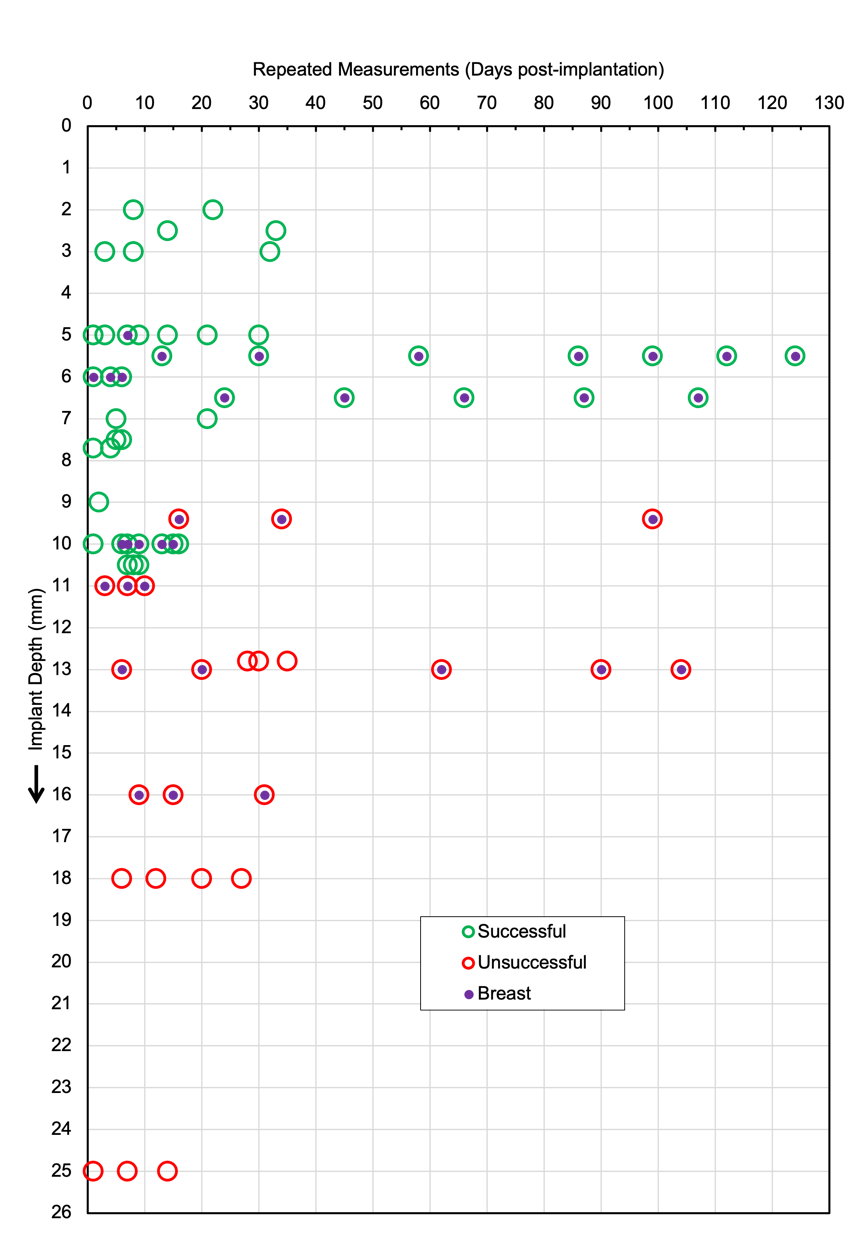
**

**Supp. Fig. 4 Effect of implant depth on EPR detection sensitivity.** The OxyChip implant depths from the skin surface were estimated by gross assessment in the pathology lab on the resected tumor and/or from the ultrasound images used for guiding the implantation. The EPR measurements were considered successful if the median spectrum showed the characteristic single-component signal centered at the expected magnetic-field-sweep position corresponding to the OxyChip signal; otherwise, the measurements were noted as unsuccessful. The plot shows the measurements outcome (successful or unsuccessful) for all 70 measurement sessions of 23 patients, excluding patient 6. None of the tumors measured showed mixed results, that is, they had either all successful or all unsuccessful measurement sessions.
